# A proteomic analysis of peanut seed at different stages of underground development to understand the changes of seed proteins

**DOI:** 10.1371/journal.pone.0243132

**Published:** 2020-12-07

**Authors:** Haifen Li, Xuanqiang Liang, Baojin Zhou, Xiaoping Chen, Yanbin Hong, Ruo Zhou, Shaoxiong Li, Haiyan Liu, Qing Lu, Hao Liu, Hong Wu

**Affiliations:** 1 State Key Laboratory for Conservation and Utilization of Subtropical Agro-Bioresources, South China Agricultural University, Guangzhou, China; 2 Crops Research Institute, Guangdong Academy of Agricultural Sciences (GAAS), South China Peanut Sub-Center of National Center of Oilseed Crops Improvement, Guangdong Key Laboratory for Crops Genetic Improvement, Guangzhou, China; 3 Technical Service Departments, Deepxomics Co., Ltd., Shenzhen, China; Jawaharlal Nehru University, INDIA

## Abstract

In order to obtain more valuable insights into the protein dynamics and accumulation of allergens in seeds during underground development, we performed a proteomic study on developing peanut seeds at seven different stages. A total of 264 proteins with altered abundance and contained at least one unique peptide was detected by matrix-assisted laser desorption ionization time-of-flight/time-of-flight mass spectrometry (MALDI-TOF/TOF MS). All identified proteins were classified into five functional categories as level 1 and 20 secondary functional categories as level 2. Among them, 88 identified proteins (IPs) were related to carbohydrate/ amino acid/ lipid transport and metabolism, indicating that carbohydrate/amino acid/ lipid metabolism played a key role in the underground development of peanut seeds. Hierarchical cluster analysis showed that all IPs could be classified into eight cluster groups according to the abundance profiles, suggesting that the modulatory patterns of these identified proteins were complicated during seed development. The largest group contained 41 IPs, the expression of which decreased at R 2 and reached a maximum at R3 but gradually decreased from R4. A total of 14 IPs were identified as allergen-like proteins by BLAST with A genome (*Arachis duranensis*) or B genome (*Arachis ipaensis*) translated allergen sequences. Abundance profile analysis of 14 identified allergens showed that the expression of all allergen proteins was low or undetectable by 2-DE at the early stages (R1 to R4), and began to accumulate from the R5 stage and gradually increased. Network analysis showed that most of the significant proteins were involved in active metabolic pathways in early development. Real time RT-PCR analysis revealed that transcriptional regulation was approximately consistent with expression at the protein level for 8 selected identified proteins. In addition, some amino acid sequences that may be associated with new allergens were also discussed.

## Introduction

As an important oilseed crop worldwide, peanut (*Arachis hypogaea* L.) is rich in proteins and fats, and it plays a crucial role in the oilseed economy of many countries [1]. Like other plant seeds, the formation of peanut seeds can be divided into three stages, including morphogenesis, seed filling and seed desiccation [2, 3]. Unlike other plants, peanut has distinctive reproductive developmental processes. Peanut flowers are produced aerially, and gynophores form after fertilization. The peanut gynophores carrying fertilized ovules grow downward until they penetrate the soil. After penetrating into the soil, the gynophore stems stop elongating, and the fruits in its tips begin to swell and mature horizontally [4, 5].

Recently, a great deal of attention has been paid to seed development, and seed development of many plants has been studied using proteomic approaches, including *Lotus japonicus* (*L*. *japonicus*) [6], *Medicago truncatula* [7–9], soybeans (*Glycine max*) [10–12] peas (*Pisum sativum* L.) [13], Masson pine (*Pinus massoniana Lamb*) [14] and so on. Consequently, many molecular modulators of seed development have been recognized in these crop plants, especially in the model plant *Arabidopsis*. Many transcription factors have been reported, including negative regulators (*AP2*, *ARF2* and *DA1*) and positive regulators (*IKU1*, *IKU2*, *MINI3*, *AGL62*, *TTG2*, *SHB1* and *KLUH*). In addition, some signaling peptides have also been shown to affect seed development, including CLE8, CRPs, ESF1, and MEG1 [2].

Although several studies have carried out transcriptomic projects using high-density oligonucleotide microarrays and RNA-seq to study the gene expression profiles during peanut seed development [15–17], peanut seed development has not yet been characterized systematically. Moreover, cellular metabolic events are also controlled by protein–protein interactions, posttranslational protein modifications and enzymatic activities that cannot be described by transcriptional profiling approaches alone. There have been several studies on peanut seed proteomics, but these studies mainly used proteomic techniques to identify the differentially expressed proteins associated with special traits [18–21]. However, little was known about protein expression and accumulation patterns, and protein interaction networks during peanut seed development.

In addition, peanut seeds contain 22–33% protein, and are currently used worldwide as a food ingredient due to their high-quality protein [22]. However, most of the peanut protein content is made up of seed storage protein, many of which are allergen proteins [25]. Peanut allergies are one of the most severe food allergies. Some people may have severe anaphylaxis after eating trace amounts of allergen proteins [23, 24]. To date, twenty proteins belonging to 7 protein families are currently classified as allergens by the WHO/IUIS Allergen Nomenclature Sub-Committee (http://www.allergen.org), the only body of experts authorized to assign official allergen designations. The problem of peanut allergens has aroused great concern [25], and there are many reports on the sequences [26, 27], structures [28], classification [29], biological functions [30, 31] and diagnosis [32] of peanut allergens. However, information related to peanut allergen types and abundance at different developmental stages, which are important for peanut food processing, is still unknown.

Here, a proteomic analysis of peanut seeds at seven distinct underground developmental stages was carried out by two-dimensional analysis, and the first proteomic map of the seed developmental proteome was drawn with the following objectives: (1) to catalog protein expression patterns during peanut seed development; (2) to characterize peanut allergen accumulation at seven developmental stages; and (3) to analyze protein–protein interaction networks. Taken together, our data could serve as a valuable resource for understanding the seed development of peanut and reveal the accumulation of allergen proteins during seed development.

## Materials and methods

### Peanut seed collection and protein extraction

The peanut cultivar Yueyou7 was obtained from Crops Research Institute, Guangdong Academy of Agricultural Sciences, China. Peanut plants were cultivated in an experimental nursery under normal conditions. The samples were subterranean peanut seeds from peanut plants at seven developmental stages. These seven developmental stages were classified according to parameters described in a previous study [17], such as pod diameter and seed diameter. The detailed sample information is shown in Table 1.

**Table 1:** Summary of peanut seed samples during pod development.

Stages	Samples	Pod size (mm)	Seed size (mm)
R1	seed	7.0–10.0	1.0–2.0
R2	seed	10.0–13.0	1.0–2.0
R3	seed	13.0–16.5	2.0–4.0
R4	seed	13.0–16.5	4.0–6.0
R5	seed	13.0–16.5	6.0–8.0
R6	seed	13.0–16.5	8.0–10.0
R7	seed	13.0–16.5	10.0–12.0

Protein isolation and 2-D gel electrophoresis were performed using the protocols described by Zhang et al. [33]. Briefly, peanut seeds (2 g) were ground to a fine powder in liquid nitrogen and rinsed using ice-cold acetone containing 10% trichloroacetic acid (TCA) until the solution became clear. Subsequently, residual TCA was eliminated by washing the samples with acetone twice. Then, the samples were air dried and extracted with phenol (90%). The isolated proteins were precipitated with 10 volumes of ice-cold methanol consisting of 0.1 M ammonium acetate. Next, the proteins were dissolved in guanidine hydrochloride (6 M) containing 5 mM tributylphosphane (TBP) and 10 mM 2-vinylpyridine (2-VP) for protein reduction and alkylation, and the alkylated proteins were reclaimed by reacting with 10 volumes of ice-cold ethanol for 2 h in the dark.

The recovered protein samples were re-dissolved in lysis buffer composed of 7 M urea, 2 M thiourea and 4% 3-[(3-Cholamidopropyl)dimethylammonio]propanesulfonate (CHAPS), and the protein concentration was determined using a Bio-Rad protein assay kit (Bio-Rad, Cambridge, MA). For isoelectric focusing (IEF), proteins (1 mg) were dissolved in a final volume of 450 μL, loaded onto nonlinear immobilized pH gradient (IPG) strips (pH 3–10, 24 cm, Bio-Rad, US) and rehydrated at 20°C for 10 h. Electrofocusing (IsoelectrlQ 2, PSL, Australia) was performed at 20°C, and the voltage was gradually increased as follows: 100 V for 1 h, from 100 to 10,000 V for 8 h and then at 10,000 V for 8 h. The IPG strips were then equilibrated in buffer [50 mM Tris-HCI pH 8.8, containing 6 M urea, 2% (wt/vol) SDS, 30% (wt/vol) glycerol and 0.1% bromophenol blue] for 15 min. SDS-PAGE was carried out using homemade 15% polyacrylamide gels (Bio-Rad PROTEAN Plus Dodeca Cell) at 50 mA/gel. For 2-D gel analysis, the protein spots were stained by colloidal Coomassie Brilliant Blue G-250. Images were acquired using a calibrated densitometer (GS-800, Bio-Rad, USA) and assessed with ImageMaster 2-D Platinum 5.0 software (Amersham Biosciences, UK).

### Analysis of the 2-D gel images

After automatic spot detection, reproducible spots were defined as the spots matching in at least two out of three gels for each sample and included in the synthetic 2-D gel images. For comparison of gels, intensity data of individual protein spots were normalized according to the Image Master Software user manual. Spots that changed at least two-fold based on statistical analysis of the 7 samples were differentially expressed proteins. P values less than 0.05 were considered statistically significant.

### Protein detection by MALDI-TOF/TOF MS

Target protein spots were manually excised from the gels and digested with trypsin (Promega) based on the manufacturer’s protocols. Digested protein extracts were re-suspended in 5 μL of 0.1% trifluoroacetic acid, and then the samples were mixed (1:1 ratio) with 50% acetonitrile-1% trifluoroacetic acid containing a saturated solution of α-cyano-4-hydroxy-trans-cinnamic acid. Subsequently, 1 μL aliquots were spotted onto stainless steel sample target plates. Mass spectra of peptides were acquired on a Sciex 5800 MALDI TOF/TOF mass spectrometer. Data were obtained in a positive MS reflector, and a CalMix5 standard was used to calibrate the instrument (ABI4700 Calibration Mixture). Mass spectra were obtained from each sample spot by accumulation of 900 laser shots within a mass range of 900–4,000 Da. For MS/MS spectra, the 12 most abundant precursor ions per sample were chosen for subsequent fragmentation, and 2,000 laser shots were accumulated per precursor ion. A minimum signal-to-noise ratio (S/N) of 60 was used as the criterion for precursor selection. Both the MS and MS/MS data were processed and directly exported to peak list files by using the script tool integrated in TOF-TOF series explore software. The following peak-picking parameters were used: MS, range from 900 to 4,000; S/N of 15; MS/MS, range from 60 to below 20 of precursor mass.

### Bioinformatic analysis

The peak list files were searched against a combined protein database with decoy sequences using Mascot software (version 2.3.1, Matrix Science, Boston, MA). The combined protein database contained the proteins from the peanut A-genome progenitor (*Arachis duranensis*) (https://www.ncbi.nlm.nih.gov/genome?LinkName=assembly_genome&from_uid=1080891, 52,826 sequences) and B-genome progenitor (*Arachis ipaensis*) (https://www.ncbi.nlm.nih.gov/genome/35711, 57,621 sequences). For protein identification, the MS/MS search parameters included a precursor mass tolerance of 100 ppm, a fragment ion mass tolerance of 0.5 Da, full cleavage by trypsin with one missed cleavage permitted, carbamidomethyl (C) as the fixed modification, and oxidation (M) as variable modifications. The Mfuzz R package was used for the statistical analysis of protein quantification. Protein functions were annotated with an online Eggnog database (http://eggnogdb.embl.de). The protein function networks were analyzed by the STRING (Search Tool for the Retrieval of Interacting Genes/Proteins) system 9.1 (http://stringdb.org/) and visualized with Cystoscope.

### Total RNA Extraction and Real-time PCR

RNA Extraction and Real-time PCR was carried out according to a previously described method [34]. The concentration and integrity of purified RNA was assessed using a UV-visible spectrophotometer, DNA NanoDrop (Thermo Fisher, Waltham, MA, USA). RNase-freeDNase I (Fermentas, Waltham, MA, USA) was used to remove genomic DNA contaminants, and 1 μg of total RNA was reversely transcribed into cDNA using a PrimeScript RT reagent Kit (Takara, Dalian, China) in accordance with the manufacturer’s instructions. First-strand cDNA was synthesized from 1 μg total RNA using the ReverTra Ace-α-First Strand cDNA Synthesis kit (TOYOBO) according to the manufacturer’s protocols. Gene-specific primers were designed with the Primer Premier 5.0 (PREMIER Biosoft International, USA). Quantitative real-time PCR was performed with Realtime PCR Master Mix (TOYOBO) and a Light Cycler 480 instrument (Roche) equipped with Light Cycler Software Version 1.5 (Roche) based on the manufacturer’s instructions. Amplification reactions were carried out in a total volume of 20 μL. The actin gene was amplified along with the target genes and used as an endogenous control to normalize expression between different samples. All assays for each particular gene were performed in triplicate synchronously under identical conditions. Relative quantification analyses of all target genes were performed using the E (Efficiency)-method from Roche Applied Science [35]. The expression level of each target gene was normalized to the level of the reference gene. The relative expression values were then validated for the 2-DE proteomic data.

## Results

### High-resolution proteomes of developing peanut seeds

**Fig 1:**
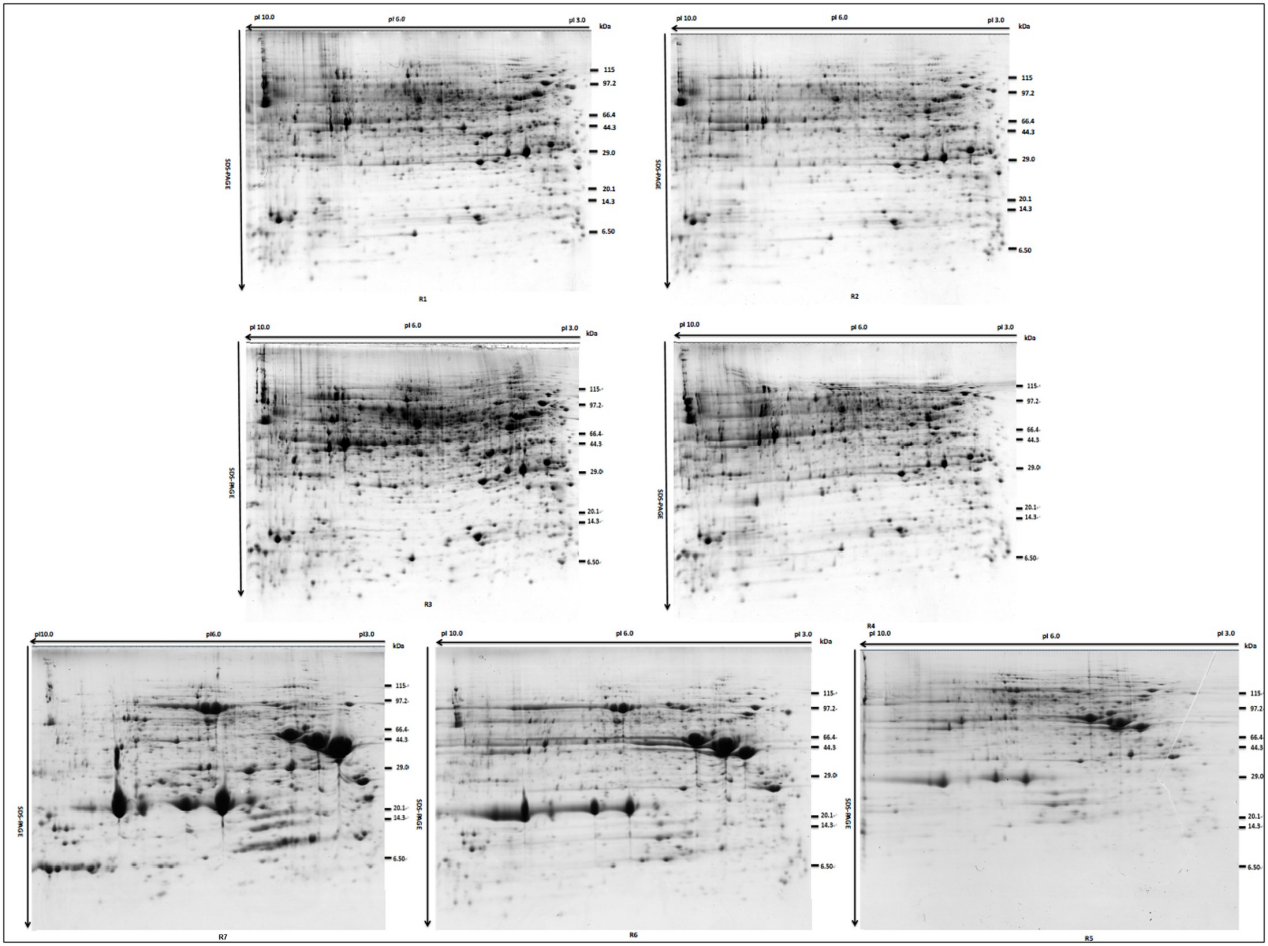
2-D gel electrophoresis analysis of proteins isolated from developing peanut seeds. Seed samples were collected at R1-R7 development stages respectively. Seed development stages were monitored by pod diameter and seed diameter (Table 1). Total proteins were separated by 2-D gel electrophoresis with IEF (pH 3 to 10), and gels were stained by Colloidal Coomassie Brilliant Blue G-250.

In order to comprehensively understand the protein expression profile of peanut seeds, developing peanut seeds at 7 developmental stages (R1-R7) (Table 1) were harvested precisely by pod size and seed size. Then, the whole proteins were resolved and detected using high-resolution two-dimensional electrophoresis (2-DE) followed by colloidal Coomassie brilliant blue staining. Each experiment was conducted in triplicate. From the 2-DE maps, it was showed that the underground development of peanut seeds can be roughly divided into the early stage of development (R1-R4) and late stage of development (R5-R7), and the proteome during peanut seed development was highly dynamic (Fig 1). Using ImageMaster 2-D Platinum 5.0, more than 800 reproducible protein spots were observed in at least three replicate gels, suggesting that they were involved in peanut seed development.

### Identification of dynamically accumulated peanut seed proteins

To select proteins that were differentially accumulated over seven developmental stages, the proteome profiles were compared using ImageMaster software, and 275 protein spots with differential staining at different development stages were detected (at least 2-fold change, p≤0.05) in combination with manual validation and quantification. Then, they were excised from the 2-DE gels and identified by MALDI-TOF/TOF-MS MASCOT and MASCOT software against a peanut protein sequence database. A total of 264 proteins that contained at least one unique peptide with 1% FDR (false discovery rate) were identified (S1 Table). Approximately 80% of the proteins were composed of more than one peptide. Proteins with molecular weights of 10–20 kDa, 20–30 kDa, 30–40 kDa, 40–50 kDa, 50–60 kDa, 60–70 kDa, 70–80 kDa, 80–90 kDa and 90–100 kDa accounted for 6.8%, 18.9%, 17.4%, 24.2%, 17.4%, 9.8%, 3.4%, 1.5% and 0.4%, respectively. The identified proteins containing 1, 2, 3, 4, 5 and more than 5 peptides accounted for 20.1%, 12.9%, 12.5%, 10.2%, 11.4% and 33% respectively.

### Functional categorization of identified peanut seed proteins

**Fig 2:**
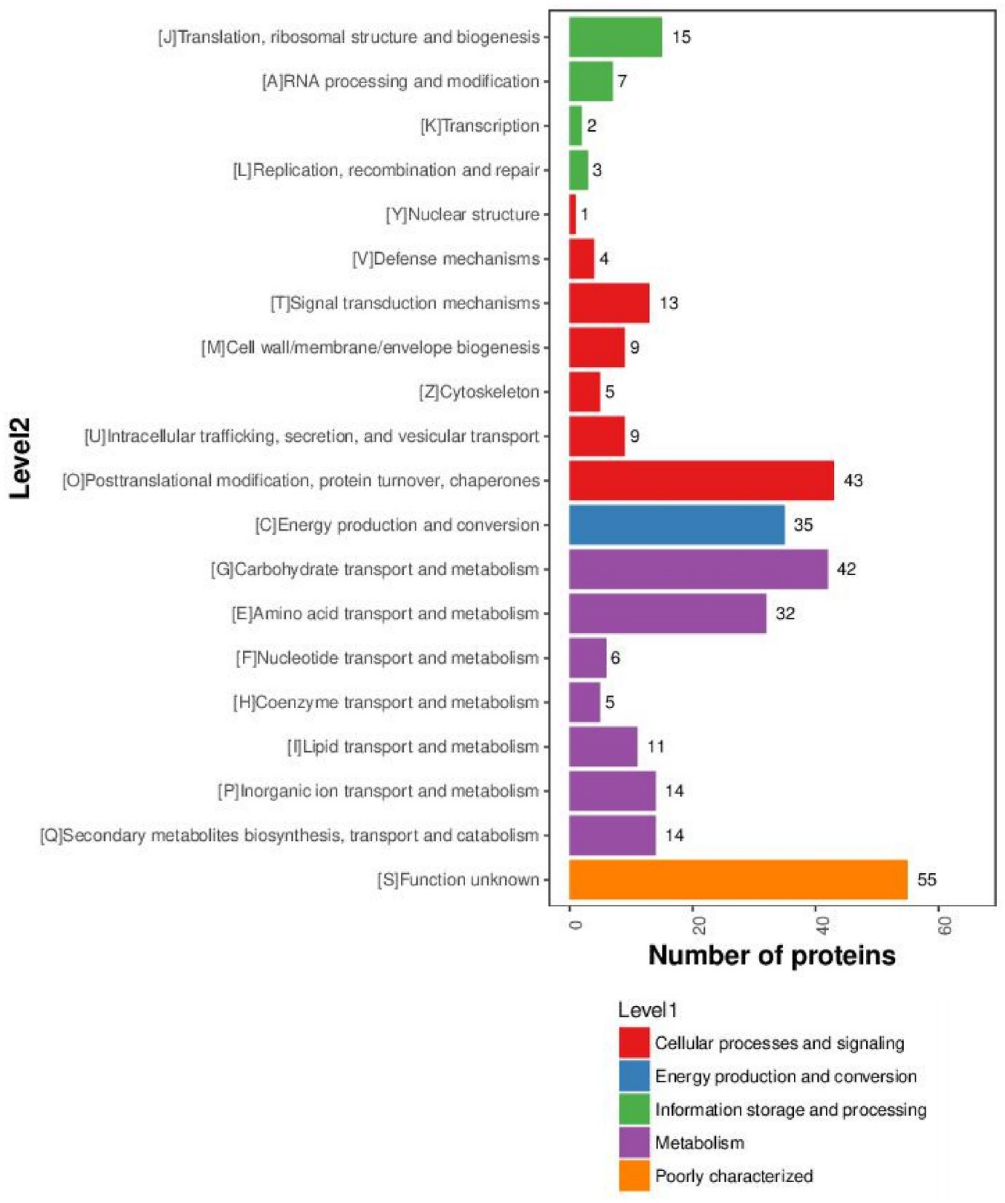
Functional categorization of all identified proteins during underground developmental stages. A total of 264 proteins were classified into 5 primary functional categories as the “Level 1” and 20 secondary functional categories as the “Level 2”.

In order to evaluate the overall functions of the identified proteins, the online EggNOG database was employed to classify the functions of all identified peanut proteins. Finally, these proteins were classified into 5 primary functional categories as level 1 and 20 secondary functional categories as level 2 (Fig 2). The 5 primary functional categories included metabolism process, cellular processes and signalling, poorly characterized, energy production and conversion, information storage and processing, and contained 124, 84, 55, 35 and 27 IPs respectively. Metabolic process, which contained the most IPs, was further categorized into carbohydrate transport and metabolism (42 IPs), amino acid transport and metabolism (32 IPs), inorganic ion transport and metabolism (14 IPs), secondary metabolites biosynthesis transport and catabolism (14IPs), lipid transport and metabolism (11 IPs), nucleotide transport and metabolism (6 IPs), and coenzyme transport and metabolism (5 IPs). Among them, 88 identified proteins were related to carbohydrate/amino acid/lipid transport and metabolism, indicating that carbohydrate, amino acid and lipid metabolism played a key role in the underground development of peanut seeds. Cellular processes and signalling, the second largest category included 84 IPs, of which posttranslational modification, protein turnover, and chaperones accounted for 43 IPs.

### Hierarchical clustering analyses of peanut seed proteins

**Fig 3:**
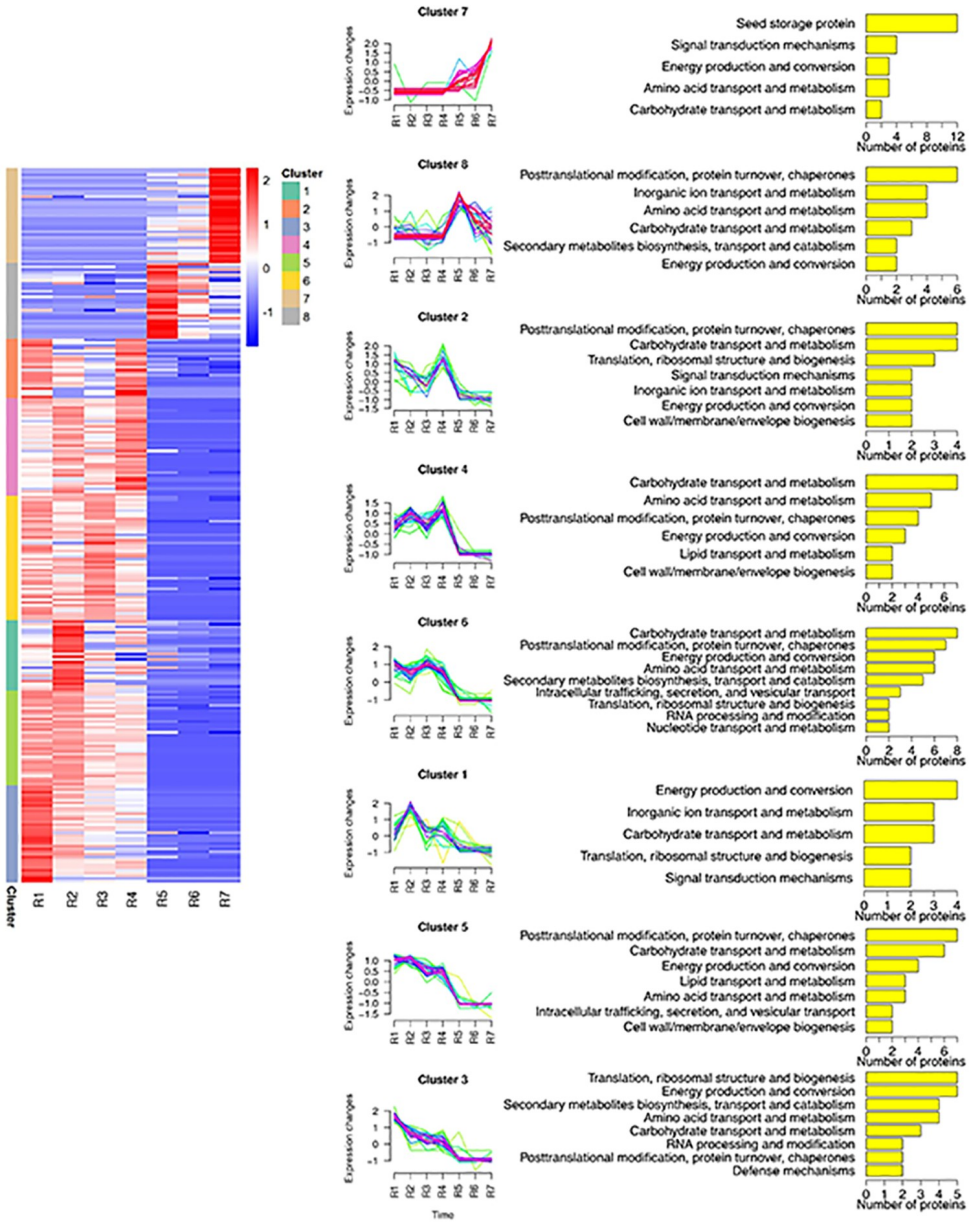
Peanut protein expression profiles during seeds development. (A) Hierarchical clustering of the identified proteins. (B) Protein expression profiles of all proteins. (C) Functional categorization of each cluster. Clustering was performed based on the protein expression levels during seven stages of seed underground development. Based on the similarity of the kinetic expression pattern, eight main clusters were generated.

From Fig 1, it was clearly observed that the proteome of stage R1-R4 and stage R5-R7 showed different patterns on the 2-D gel, and the analysis of the heat map also showed similar results (Fig 3). For a better understanding of the identified proteins, hierarchical clustering was employed, and the abundance profiles of the identified proteins were analyzed. All identified proteins were classified into eight cluster groups (C1, C2, C3, C4, C5, C6, C7 and C8), suggesting that the modulatory patterns of these identified proteins were complicated during seed development (Fig 3). The largest group contained 41 proteins (C6), the expression of which decreased at R2 and reached a maximum at R3 but gradually decreased from R4. The second group included C3 and C5, and both clusters had 27 proteins and similar expression patterns. The expression of proteins in C3 and C5 reached a peak at R1 and then gradually declined, but the expression of proteins in C5 increased in R2. The expression of the 8 protein clusters remained at a lower level at the early stages (R1-R4) and was increased to the maximum value at R5 and then decreased gradually. The smallest cluster, C1, consisted of 14 proteins that displayed M-type expression profiles. Cluster 2 and cluster 4 contained 19 and 27 proteins, respectively. The highest expression value of the two clusters was observed at R4. Interestingly, seed storage proteins were only detected in cluster 7, and the expression sharply increased after R4. Proteins involved in the category of defense mechanisms were found only in cluster 3, showing that proteins associated with defense mechanisms were gradually downregulated with the development of seeds.

### Protein identification of peanut allergens and abundance profile analysis

**Fig 4:**
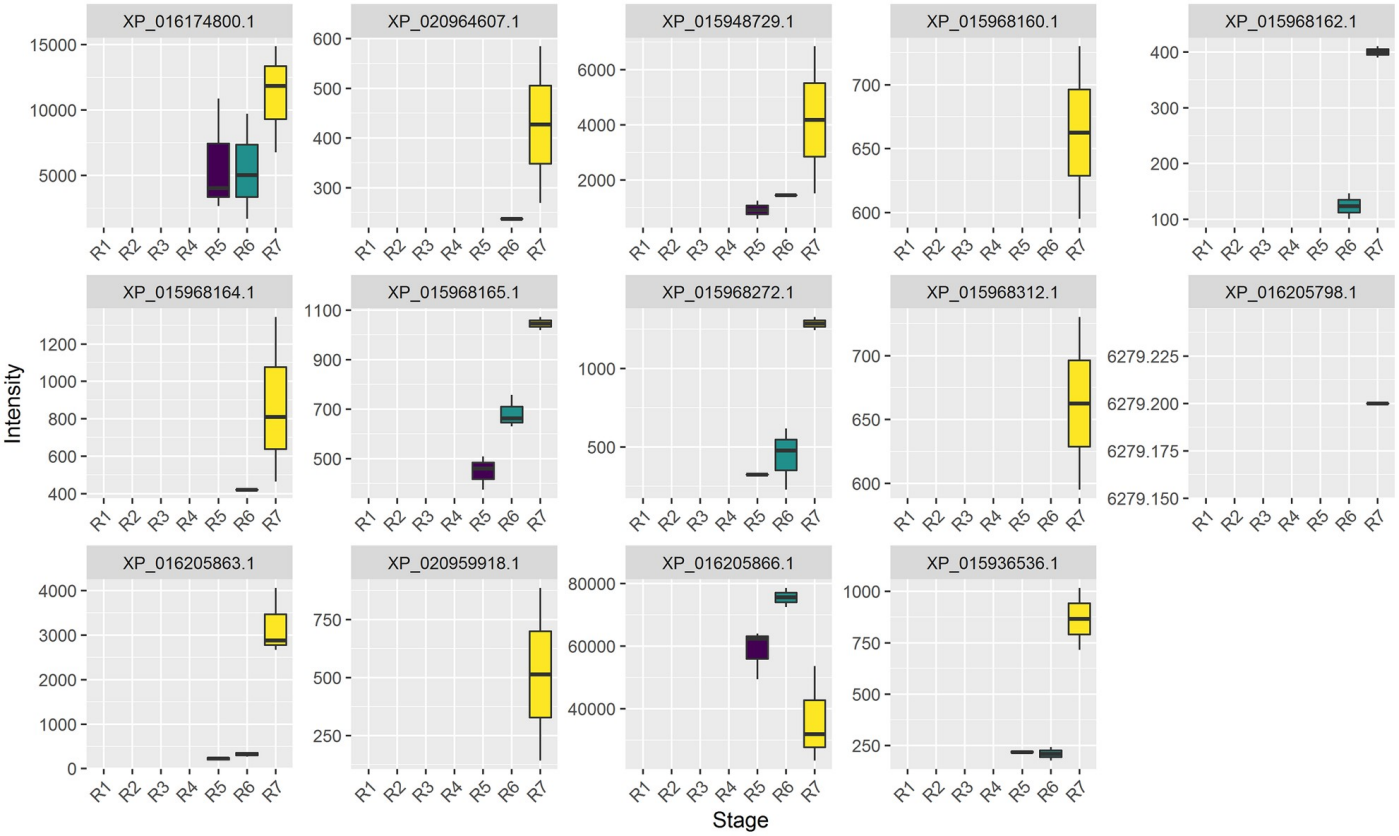
Abundance profiles of the identified allergen proteins at each developmental stage.

As one of most serious life-threatening food sensitivities, peanut allergy is becoming increasingly prevalent, particularly among children [36, 37]. In order to investigate allergen expression during seed development, allergen proteins need to be identified. In this paper, the newly published A genome (*Arachis duranensis*) and B genome (*Arachis ipaensis*) translated allergen sequences were used as criteria to identify allergens. As a result, a total of 14 proteins were identified as allergen-like proteins with the BLAST method (Table 2). To characterize the abundance profiles of allergen proteins in different stages, the expression abundance of the identified allergen in each developmental period was analyzed by summing the protein abundance, which was expressed as a relative volume. As shown in Fig 4, the relative abundance analysis of different identified allergens showed that all identified allergens from the R1 to R4 stages had low expression/accumulation or were undetectable by 2-DE. Six allergen proteins, XP_015936536.1, XP_015948729.1, XP_015968272.1, XP_015968165.1, XP_016174800.1, and XP_016205863.1, began to accumulate from the R5 stage and gradually increased. XP_015968162.1, XP_015968164.1 and XP_020964607.1 continued to accumulate from the R6 stage and had the highest abundance at the R7 stage, while XP_015968160.1, XP_015968312.1, XP_016205798.1 and XP_020959918.1 accumulated at the R7 stage. Generally, most of these allergen proteins acted as seed storage proteins (Table 2) and were assigned to cluster 7 (Fig 3), which began to accumulate in anaphase during seed development.

**Table 2:** Peanut allergen information on the differentially expressed proteins in this study.

Protein	Is allergen	Mfuzzy cluster	Mass	No. Peptides	Description	eggNOG_L1	eggNOG_L2
XP_016174800.1	Ara1	7	70997.7	11	allergen Ara h 1, clone P41B [*Arachis ipaensis*]	[P]Inorganic ion transport and metabolism;	[P] Vicilin-like antimicrobial peptides;
						[S]Function unknown	[S] nutrient reservoir activity;
							[S] Seed storage protein;
XP_020964607.1	Ara2-like	7	19641.3	1	conglutin-7 isoform X2 [*Arachis ipaensis*]	-	-
XP_015948729.1	Ara3/4-like	7	71025.9	12	legumin type B isoform X1 [*Arachis duranensis*]	[S]Function unknown	[S] seed storage protein
XP_015968160.1	Ara3/4-like	7	60715.0	11	arachin Ahy-3 [*Arachis duranensis*]	[S]Function unknown	[S] seed storage protein
XP_015968162.1	Ara3/4-like	7	58624.9	11	arachin Ahy-3 [*Arachis duranensis*]	[S]Function unknown	[S] seed storage protein
XP_015968164.1	Ara3/4-like	7	50626.0	6	legumin type B [*Arachis duranensis*]	[S]Function unknown	[S] seed storage protein
XP_015968165.1	Ara3/4-like	7	51180.2	14	arachin Ahy-3 [*Arachis duranensis*]	[S]Function unknown	[S] seed storage protein
XP_015968272.1	Ara3/4-like	7	46831.1	5	11S globulin subunit beta [*Arachis duranensis*]	[S]Function unknown	[S] seed storage protein
XP_015968312.1	Ara3/4	7	60500.8	13	arachin Ahy-3 [*Arachis duranensis*]	[S]Function unknown	[S] seed storage protein
XP_016205798.1	Ara3/4-like	7	60338.7	16	arachin Ahy-3 [*Arachis ipaensis*]	[S]Function unknown	[S] seed storage protein
XP_016205863.1	Ara3/4-like	7	59814.4	16	arachin Ahy-3 [*Arachis ipaensis*]	[S]Function unknown	[S] seed storage protein
XP_020959918.1	Ara3/4-like	7	52311.0	7	arachin Ahy-3-like [*Arachis ipaensis*]	[S]Function unknown	[S] seed storage protein
XP_016205866.1	Ara3/4-like	8	61459.2	15	arachin Ahy-3 [*Arachis ipaensis*]	[S]Function unknown	[S] seed storage protein
XP_015936536.1	Ara6	7	16909.9	3	conglutin [*Arachis duranensis*]	-	-

### Network analysis of identified proteins

**Fig 5:**
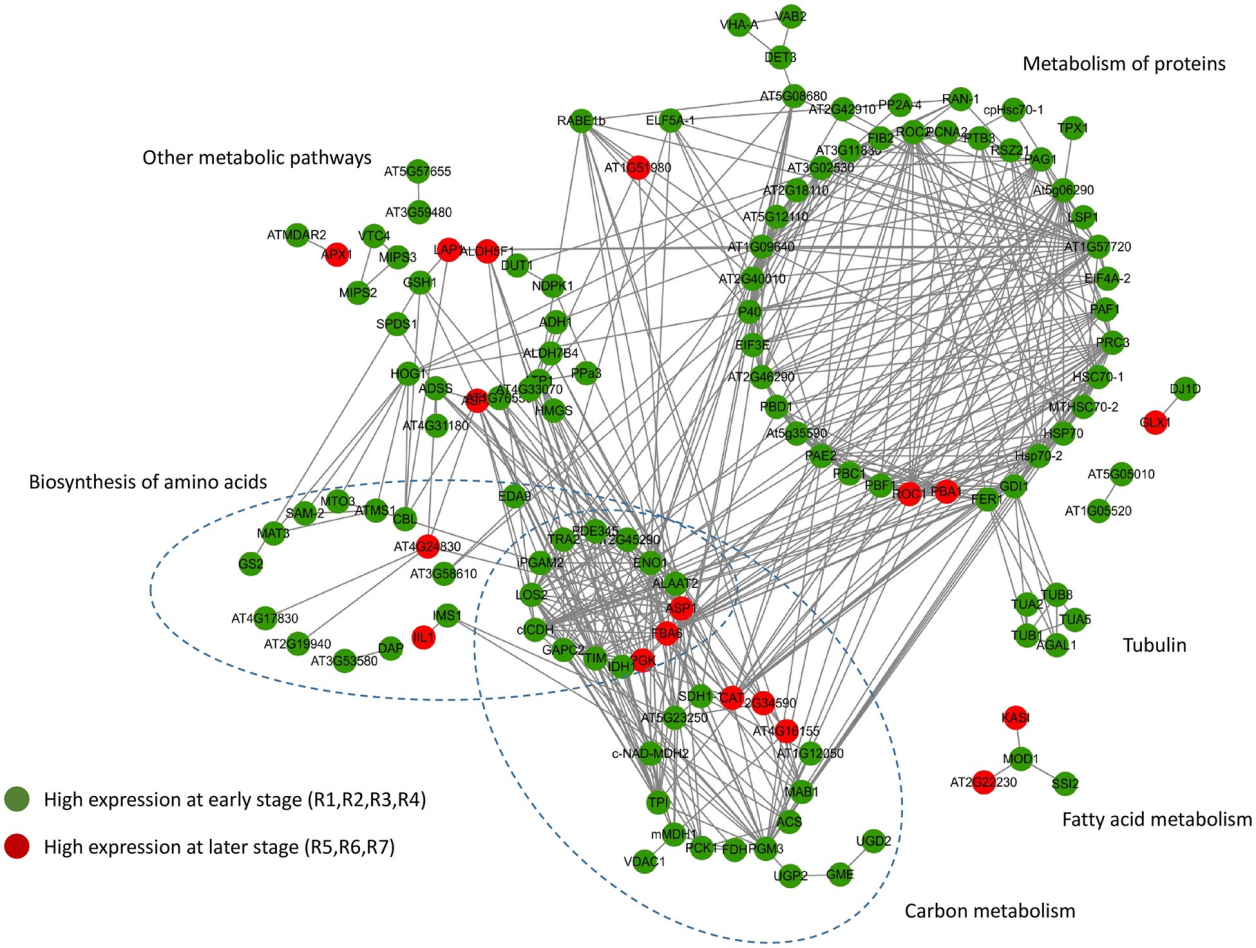
Protein-protein interaction networks of identified proteins. (Green and red indicate high expression at the early stage and late stage, respectively).

To obtain novel insight into the interrelated biological processes involved in peanut seed development, we performed network analysis using STRING integrated with GO annotation, KEGG pathway and Reactome pathway information with a high confidence (0.700) setting. The results showed that most significant proteins were involved in active metabolic pathways in early development, including metabolism of proteins, biosynthesis of amino acids, carbon metabolism and fatty acid metabolism (Fig 5). In the protein metabolism pathway, a cluster of proteasomes was enriched, including At5g35590 (XP_016181042.1), PAE2 (XP_016175801.1), PBC1 (XP_016203278.1), PBF1 (XP_016184215.1), PED1 (XP_016189206.1), and PRC3 (XP_016169678.1). In the biosynthesis of amino acids, a group of amino acid synthetases was identified, including glutamine synthetase (GS2, XP_016197383.1), S-adenosylmethionine synthetase (SAM-2, XP_016205701.1 and MTO3, XP_015945019.1), argininosuccinate synthase (AT4G24830, XP_015961120.1) and 2-isopropylmalate synthase (IMS1, XP_020966519.1).

### Analysis of expressions of identified proteins at the mRNA level

**Fig 6:**
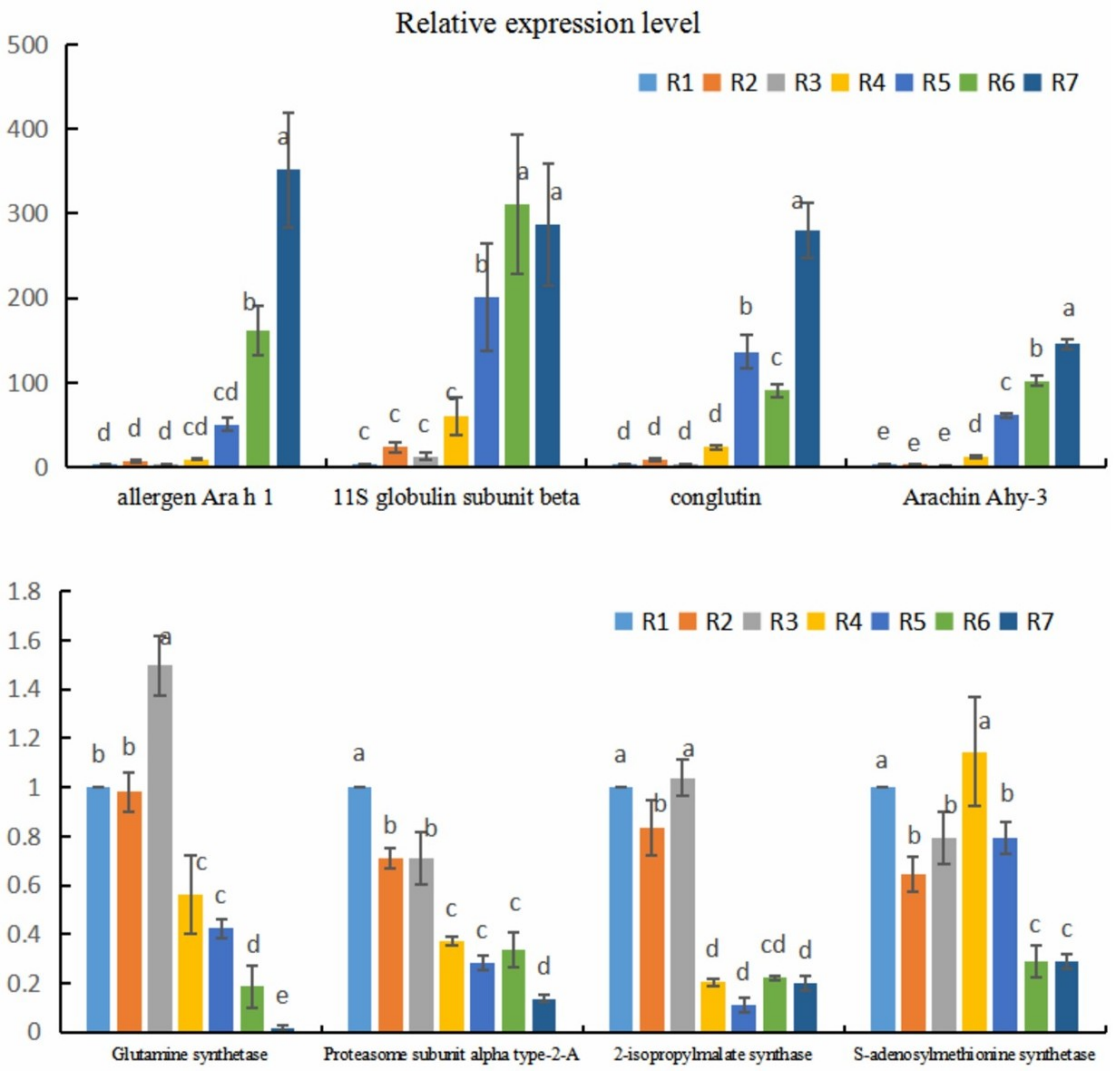
Relative transcription levels of 8 selected expressed proteins at 7 developmental stages. Actin gene was used as the internal control. Each measurement was carried out in triplicate with three biological replicates. Different lowercase letters indicate significant differences among the different accessions (t-test, p<0.05).

To validate protein expression profiling in this study, real time RT-PCR analysis were employed for the assessment of 8 differentially expressed proteins including allergen Ara h 1, 11S globulin subunit beta, conglutin, Arachin Ahy-3, glutamine synthetase, proteasome subunit alpha type-2-A, 2-isopropylmalate synthase and S-adenosylmethionine synthetase at the mRNA level. The primers were designed according to the corresponding mRNA sequences of the proteins identified by MALDI-TOF-MS analysis and the actin gene was chosen as internal control. The results (Fig 6) demonstrated that the relative expression levels of allergen Ara h 1, 11S globulin subunit beta, conglutin and Arachin Ahy-3 were lower in early stages of seed development (R1-R4), and then significantly increased in late stages (R5-R6). On the contrary, the relative expression levels of glutamine synthetase, proteasome subunit alpha type-2-A, 2-isopropylmalate synthase and S-adenosylmethionine synthetase were higher in early stages (R1-R4) and then continuously reduced at the stage of seed maturation (R4-R7). The expression of 8 selected identified proteins at the mRNA level was approximately consistent with the proteomic results and indicated that these proteins expression regulation may occurred at the transcriptional level.

## Discussion

Seed development plays an important role in the life cycle of peanuts. Peanut seeds are an important food for humans and animals because they are rich in fat and protein. The main objective of this study was to understand the accumulation of proteins in peanut seeds, especially peanut allergens, at different stages of development. These data will help elucidate the biochemical and molecular processes underlying seed filling in peanut species.

### Proteins associated with metabolism during seed development

Since peanut seeds develop underground, photosynthetic products generated by aboveground plants need to be transported underground for seed development [38]. Therefore, sucrose, the main product of photosynthesis, needs to be metabolized and transformed to sustain seed growth. In the present work, many proteins were classified into the category of metabolic process. These metabolism-related proteins were further categorized into carbohydrate transport and metabolism, amino acid transport and metabolism, inorganic ion transport and metabolism, lipid transport and metabolism, nucleotide transport and metabolism, and coenzyme transport and metabolism. These proteins exhibited various accumulation patterns during seed development. Among them, carbohydrate, amino acid and lipid metabolism accounted for 70.9%. In particular, carbohydrate transport and metabolism were distributed in each kinetic expression pattern. Carbohydrates are the main energy-supplying substances for plant cells, and carbohydrate transport and metabolism play a crucial role in the development of plants [39]. Peanut is a leguminous plant; the contents of essential amino acids are relatively high in peanut seeds, and these essential amino acids accumulate in the cotyledons as storage proteins [39, 40]. Storage protein synthesis in plant seeds is dependent on the N state, which may be controlled by amino acid transport and metabolism [41, 42]. Amino acid metabolism has been closely associated with carbohydrate metabolism since compounds derived from carbohydrate metabolism are consumed in the biosynthesis of amino acids [43].

Many metabolism-related proteins were identified in this paper. Most of these proteins were highly expressed in stages R1 to R4 and low expressed at low levels or not expressed in stages R5 to R7, especially chloroplastic proteins or chloroplastic enzymes. We hypothesize that carbohydrate and amino acid metabolism in early stages provides the raw material and energy for later peanut fruit enrichment. Chloroplasts collapse during seed maturation, and aging, chloroplastic proteins or chloroplastic enzymes are degraded. Moison et al. [36] also found that the expression of chloroplastic glutamine synthetase leaf isozyme decreases due to chloroplast degradation during plant aging. Several chloroplastic proteins have been reported to exert effects during seed development in many species. Stein et al. [37] found decreased citric acid cycle metabolites and reduced fatty acid metabolism in the frk6/frk7 double mutant of *Arabidopsis*. As a key enzyme in plants, fructose 1,6-bisphosphate aldolase (FBA) participates in glycolysis, gluconeogenesis, and the Calvin cycle. Overexpression of SlFBA7 enhances the expression and activities of other main enzymes in the Calvin cycle, leading to an increased net photosynthetic rate (Pn), seed size and stem diameter [44]. Duan et al. [45] have shown that carbon metabolism during grain filling in rice is regulated by pyrophosphate: fructose-6-phosphate 1-phosphotransferase (PFP). Moreover, a key reaction in glycolysis is catalyzed by the cytosolic enzyme glyceraldehyde-3-phosphate dehydrogenase (GAPC), which plays critical roles in cellular metabolism and seed oil accumulation [46]. Furthermore, the interconversion of glucose 1-phosphate and glucose 6-phosphate is catalyzed by phosphoglucomutase (PGM), which exists as isoforms of plastidial (pPGM) and cytosolic (cPGM). The absence of cPGM activity leads to a greatly decreased growth compared with wild-type plants, which has been evidenced by reduced rosette fresh weight, short roots, and impaired seed production [47].

### Identification of peanut allergen protein

Twenty peanut allergen proteins have been identified so far, and the corresponding amino acid sequence can be found in the UniProt database (S2 Table). After comparing the amino acid sequences of 20 allergens from the database with those translated by the A genome and B genome published recently, a total of 13 of the 20 allergens were mismatched. The cause of the mismatch is not yet clear and may be associated with the sequences [26], structures [48] and posttranslational modifications [49] of peanut allergen.

**Fig 7:**
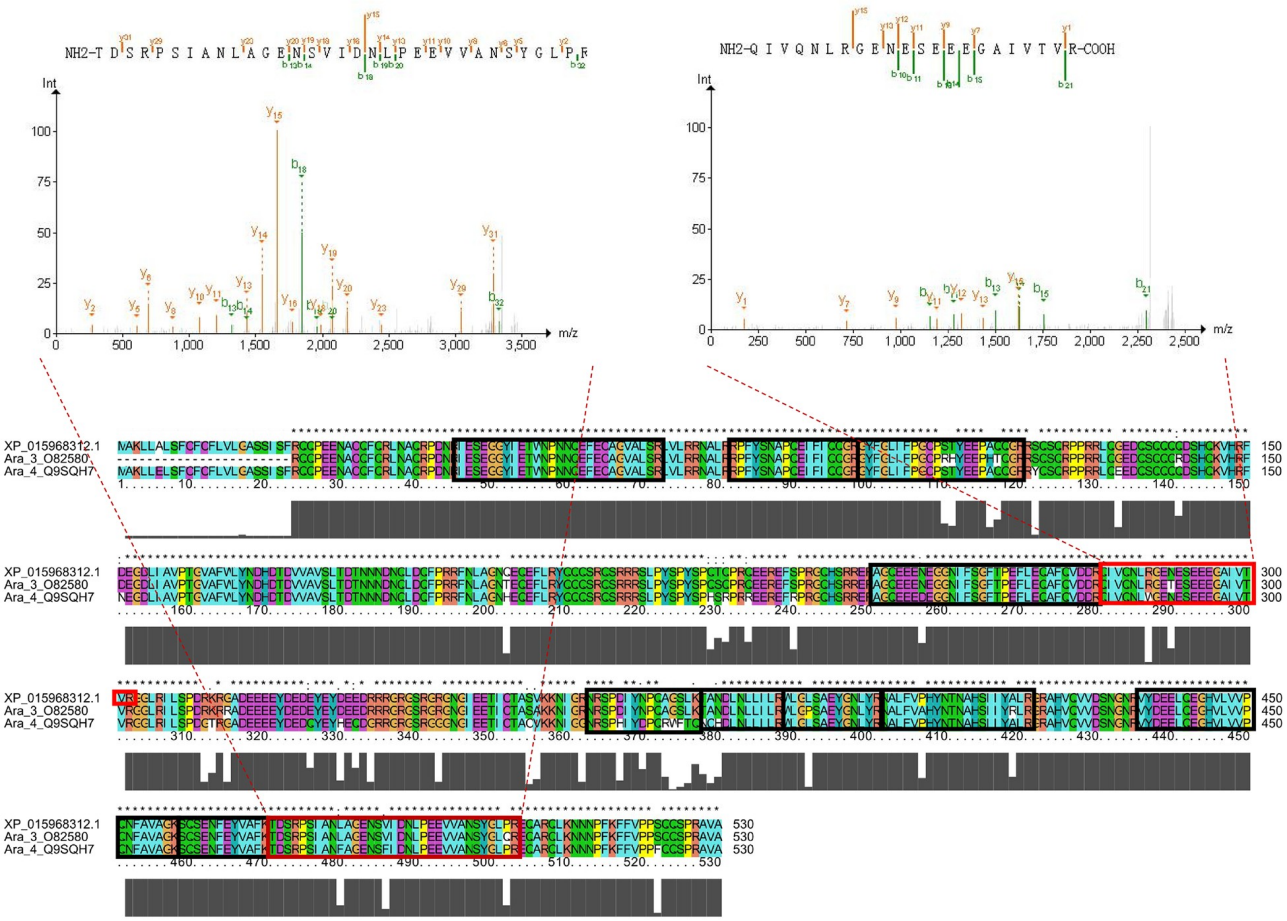
The newly identified allergen protein XP_015968312.1. (A) PSM of the new peptide KSPDEEEEYDEDEYAEEER in XP_015968312.1. (B) PSM of the new peptide AGQEQENEGGNIFSGFTSEFLAQAFQVDD in XP_015968312.1. (C) Sequence alignment of XP_015968312.1, Ara h3 and Ara h4. Unique identified peptides in XP_015968312.1 are labelled in red boxes, and the previously identified peptides are labelled in black boxes.

In this paper, the amino acid sequences of all identified proteins were BLASTed with those of the translated proteins, and only three allergic proteins, XP_016174800.1 (Ara 1), XP_015968312.1 (Ara3/Ara4) and XP_015936536.1 (Ara 6), were matched. With the adjusted criteria of Evalue < 1E-5, 11 allergen-like proteins were identified. Among them, ten proteins were defined as Ara3/4-like proteins, and most of them were annotated as arachin Ahy-3 in the A genome (*Arachis duranensis*) or B genome (*Arachis ipaensis*). The amino acid sequences of 14 identified allergens were compared with those of known allergens from UniProt, and some allergens had specific amino acid sequences. For example, XP_015968312.1, which was annotated as arachin Ahy-3 in the NCBI database, was similar to both Ara 3 (UniProt ID: O82580, bit score = 762) and Ara h4 (UniProt ID: Q9SQH7, Evalue = 0, bit score = 771) (Fig 7), and the identified peptides QIVQNLRGENESEEEGAIVTVR and TDSRPSIANLAGENSVIDNLPEEVVANSYGLPR were unique to this protein (Fig 7). XP_015936536.1, which was annotated as conglutin, was similar to Ara 6 (UniProt ID: Q647G9, E value = 1.39E^-9^, bit score = 260), and the identified peptide CCDELDQMENTER was unique to XP_015936536.1 (S1 Fig). In addition, the results for both XP_016174800.1 and XP_020964607.1 were similar (S1 Fig). Whether these proteins are new allergen proteins needs to be further verified, but we believe that such peptide information is very important for the further detection of peanut allergens.

### Abundance profiles of allergens at different developmental stages

Peanut seeds contain many allergens that are able to induce the production of specific IgE antibodies in predisposed individuals. Much effort has been focused on eliminating or reducing the effects of allergens. Thermal processing has been shown to affect peanut allergens in different manners. Compared to roasting and chemical modifications, boiling of peanuts can decrease the IgE binding capacity of Ara h 1, Ara h 2, and Ara h 3, decreasing peanut allergenicity and the IgE-binding capacity of peanut allergens [50]. Although boiled peanuts had reduced allergenicity, they could not be regarded as hypoallergenic peanuts [51]. In this paper, the allergen abundance of peanut seeds was found to be related to the maturity of peanut seeds. The greater the seed maturity was, the higher the seed allergy content. In the early stage of seed development (R1-R4), all allergens in peanuts were low or not detected. At stage R5-R6, although major allergies were also detected, their abundances were only half those at stage R7 or even lower. The results of transcriptional expression analysis also showed that 4 allergen proteins were lower in early stages of seed development (R1-R4), and significantly increased in late stages (R5-R6). Therefore, the results of this study suggest that peanuts with low maturity can be used in the production of peanut food for children and allergen-sensitive populations, which can greatly reduce the risk of peanut allergens.

## Supporting information

S1 Raw images Original, uncropped and minimally adjusted images. (PDF)

S1 Fig Details of other newly identified allergen proteins. (TIF)

S1 Table Differentially expressed proteins and the sequences of peptides identified using MALDI TOF-TOF MS. (XLS)

S2 Table Known allergen protein sequences downloaded from the UniProt database. (XLS)
