# Supplementary figures and images for: A proteomic analysis of peanut seed at different stages of underground development to understand the changes of seed proteins
Source: PLoS One. 2020 Dec 7;15(12):e0243132. doi: 10.1371/journal.pone.0243132 (PMC7721164; doi:10.1371/journal.pone.0243132)

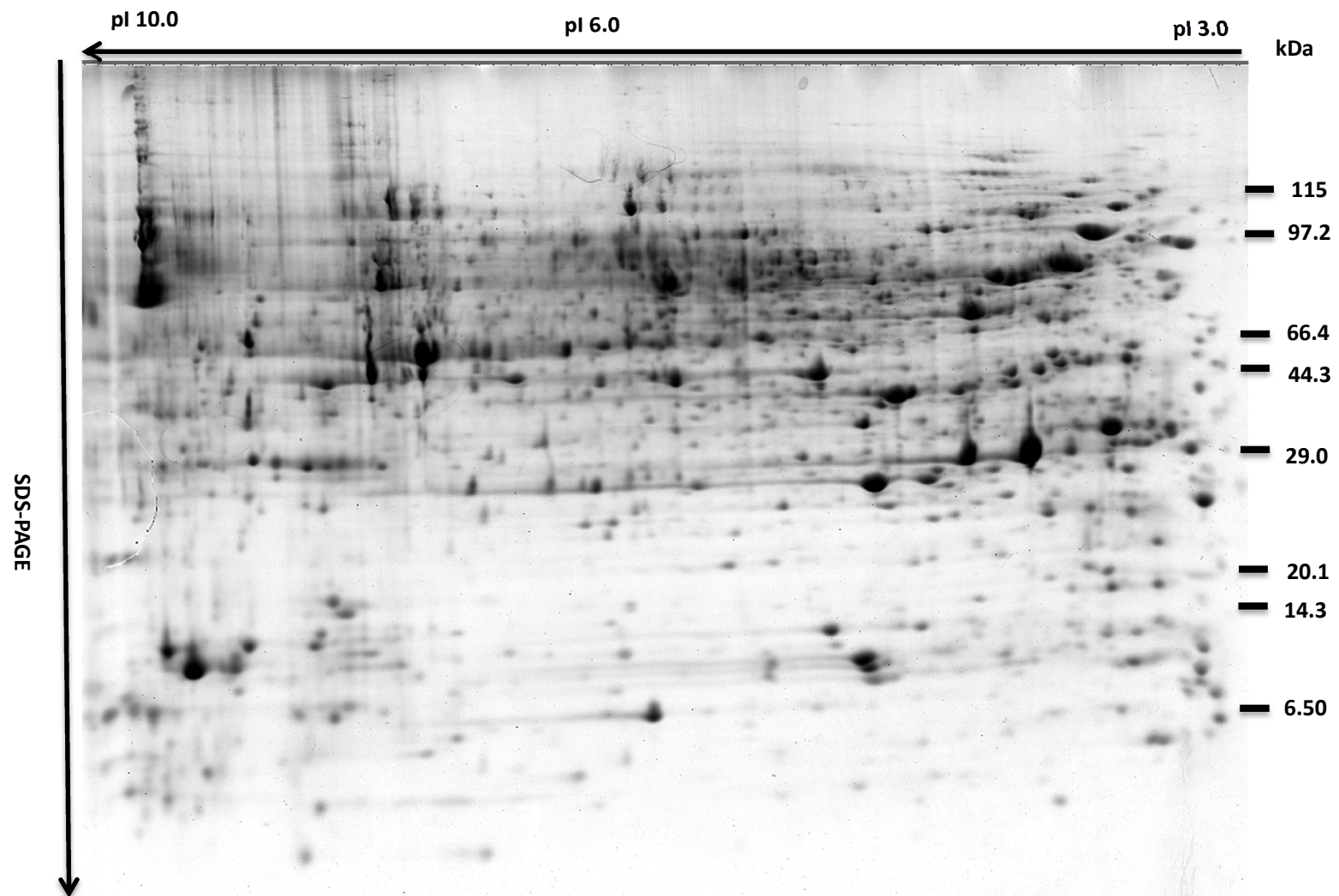

R1

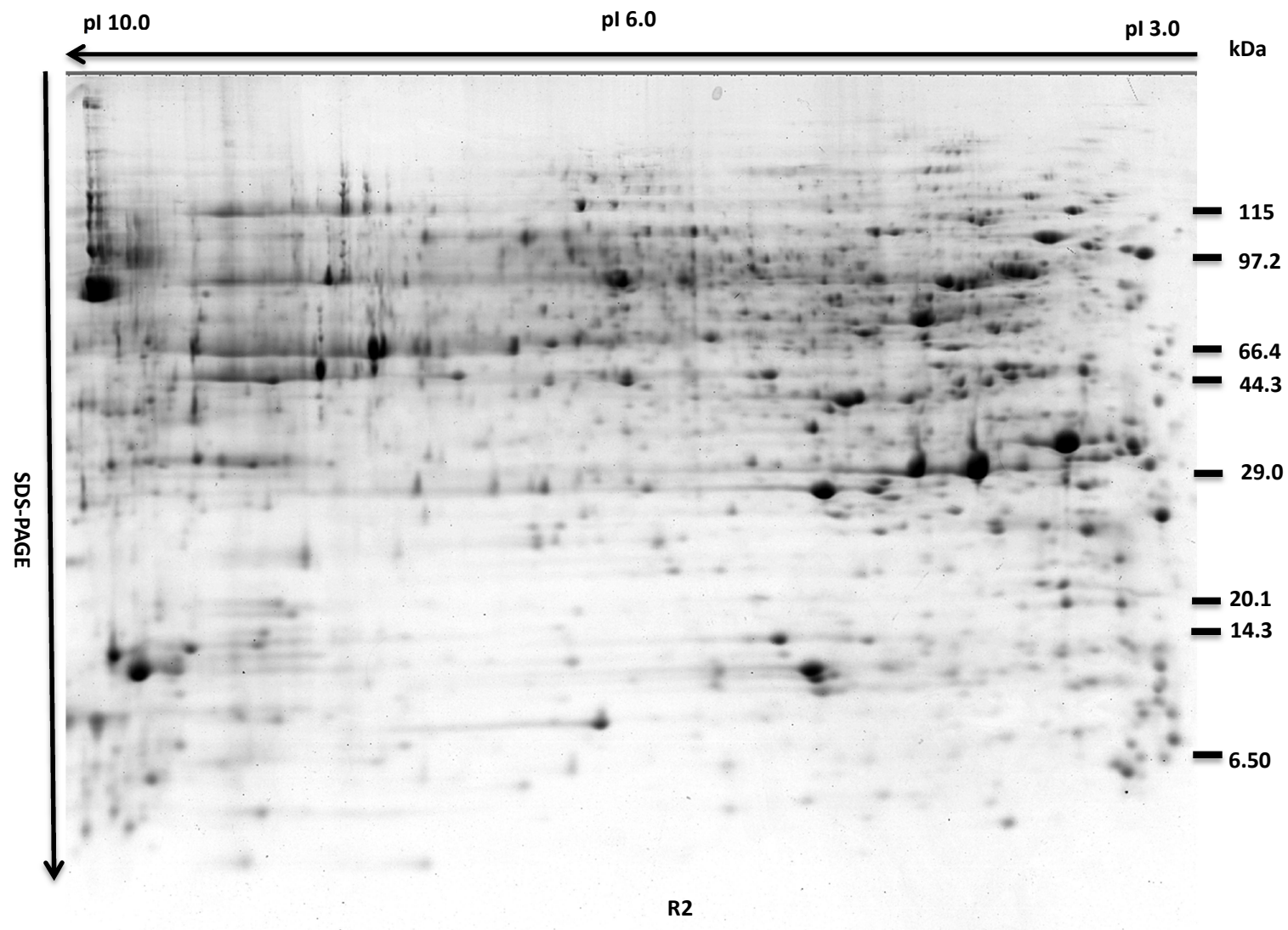

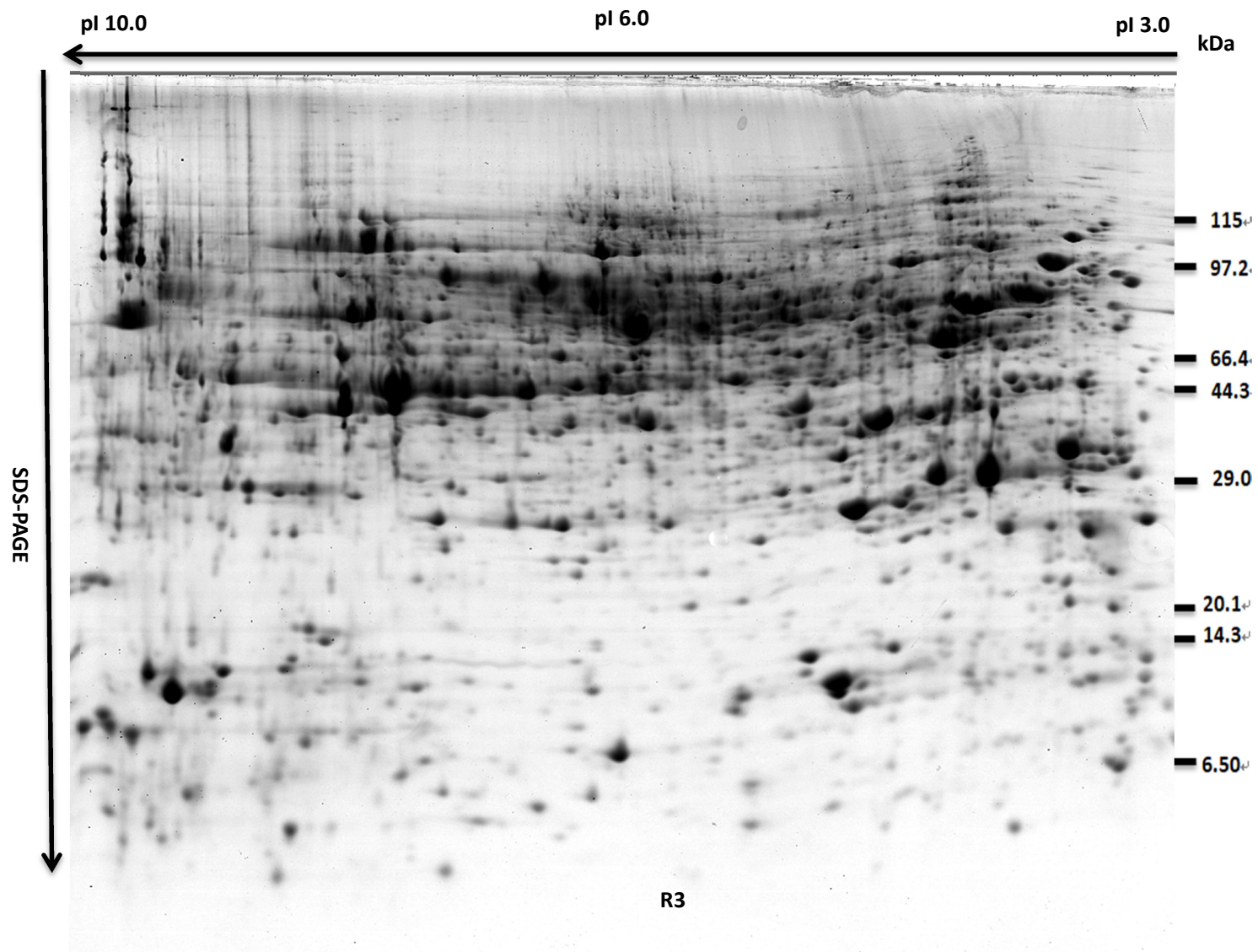

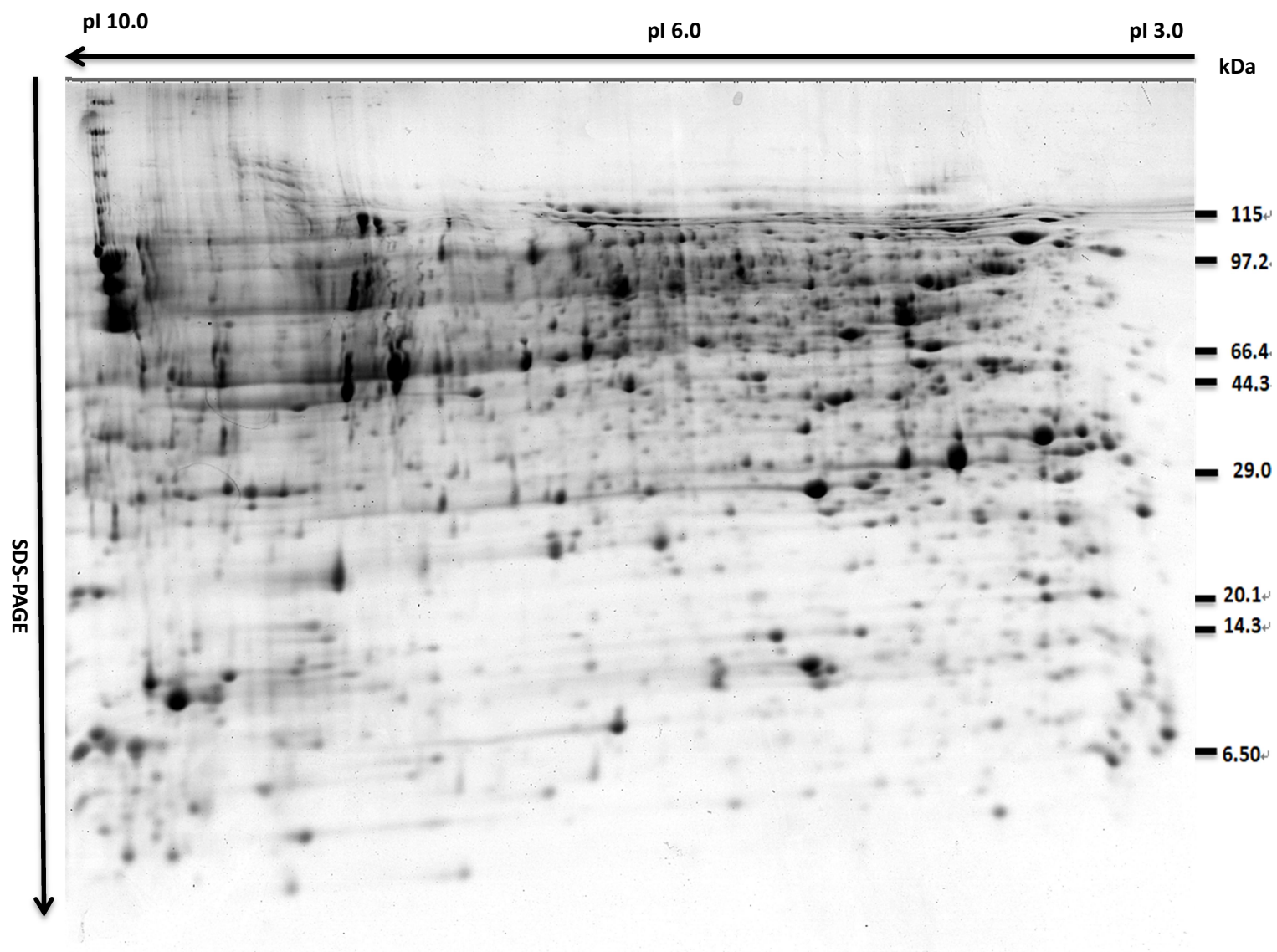

R4

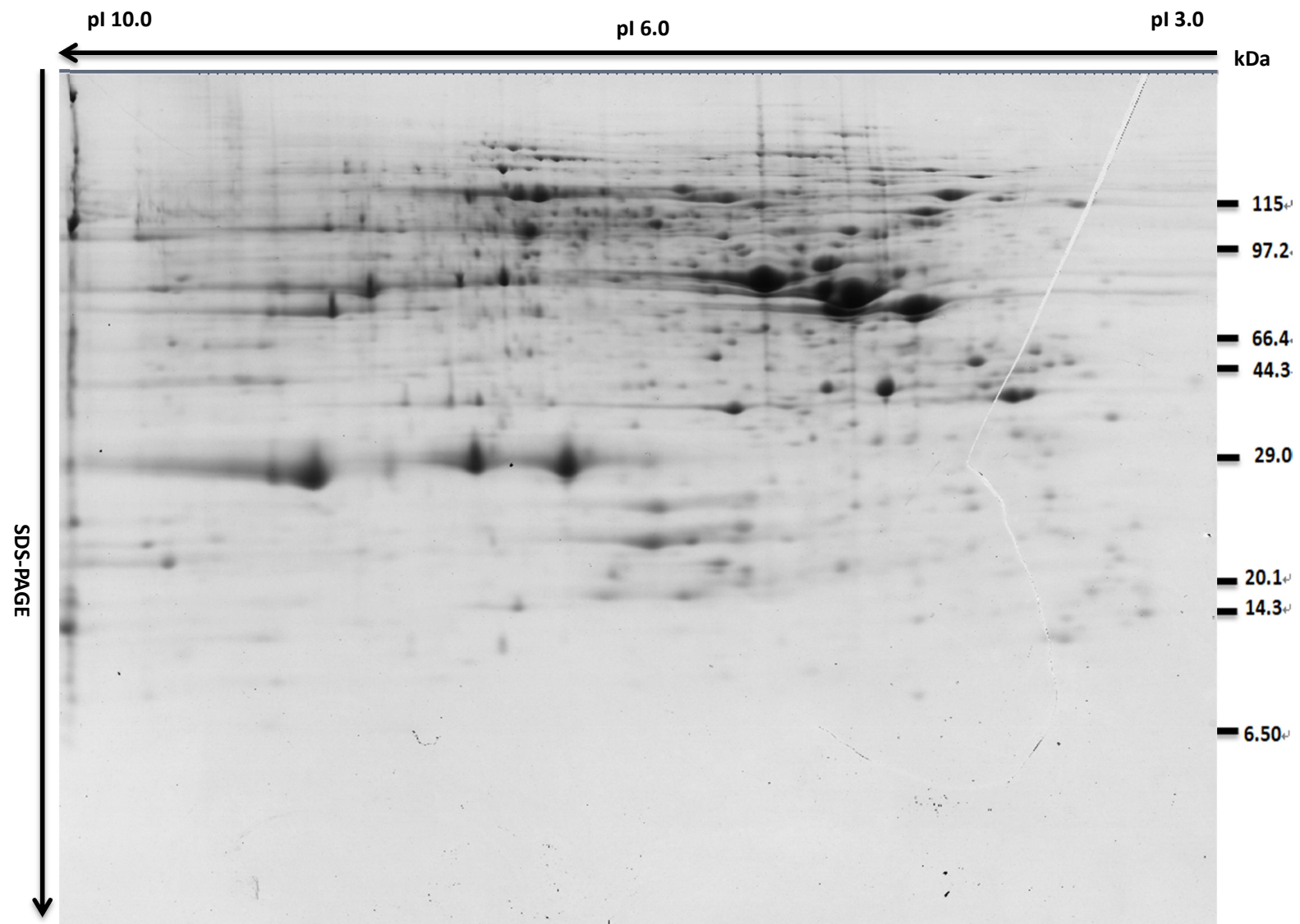

R5

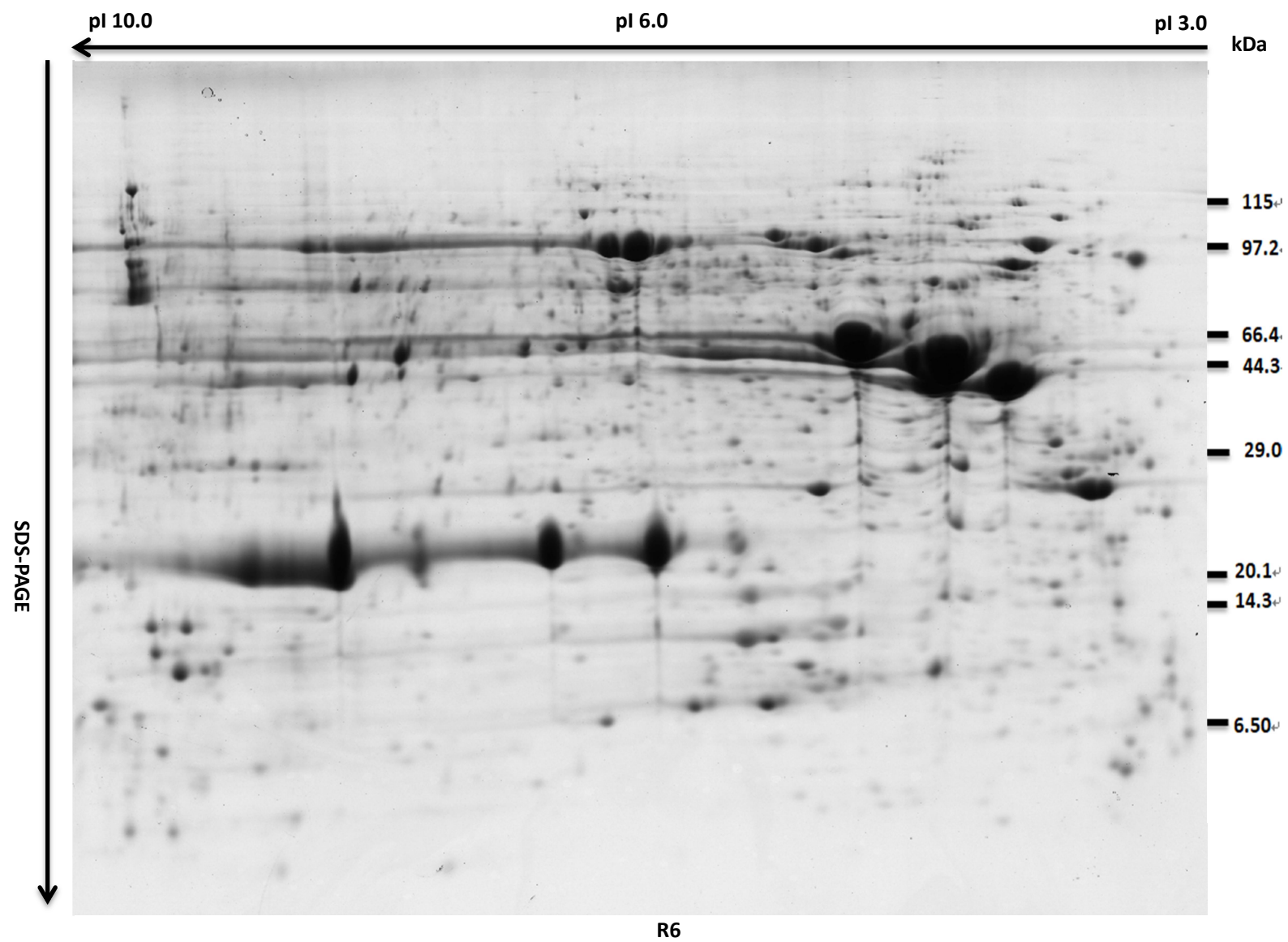

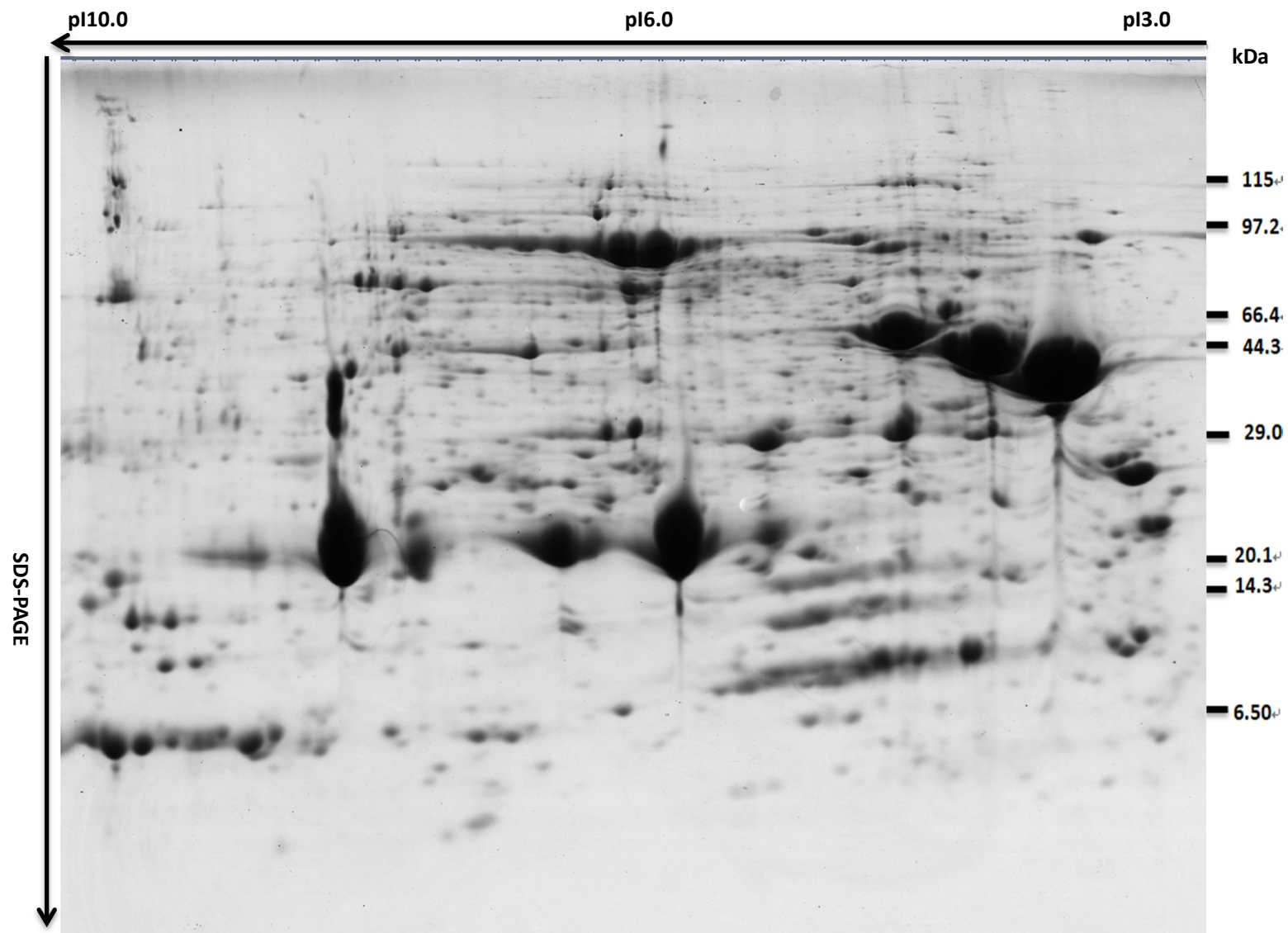

R7

Supplement: S1 Raw images — (PDF) [file pone.0243132.s001.pdf]

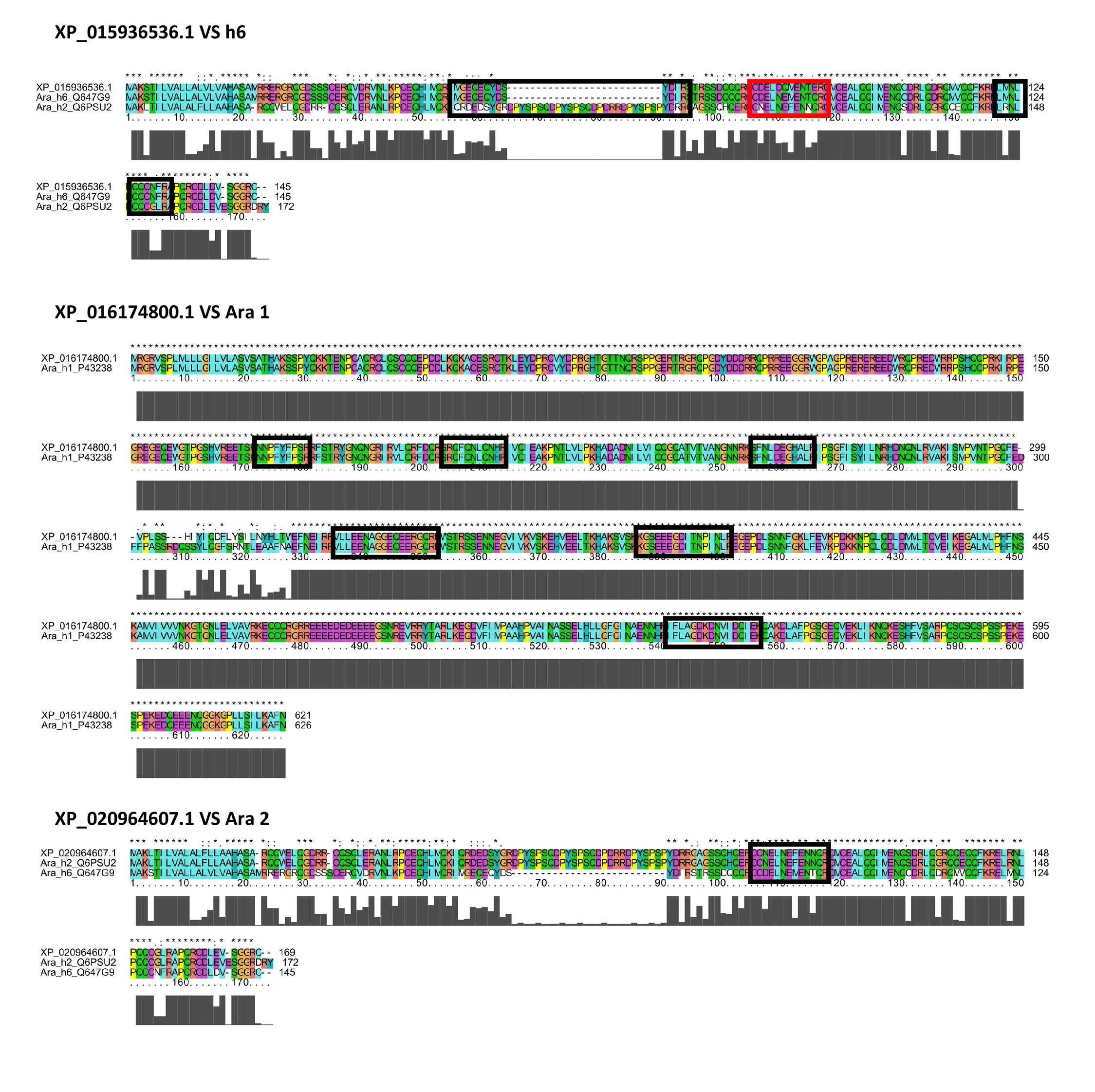

Supplement: S1 Fig — (TIF) [file pone.0243132.s002.tif]
